# Methods for the guideline-based development of quality indicators--a systematic review

**DOI:** 10.1186/1748-5908-7-21

**Published:** 2012-03-21

**Authors:** Thomas Kötter, Eva Blozik, Martin Scherer

**Affiliations:** 1 Department of Primary Medical Care, University Medical Center Hamburg-Eppendorf, Hamburg, Germany; 2 Institute for Social Medicine, University of Lübeck, Lübeck, Germany

## Abstract

**Background:**

Quality indicators (QIs) are used in many healthcare settings to measure, compare, and improve quality of care. For the efficient development of high-quality QIs, rigorous, approved, and evidence-based development methods are needed. Clinical practice guidelines are a suitable source to derive QIs from, but no gold standard for guideline-based QI development exists. This review aims to identify, describe, and compare methodological approaches to guideline-based QI development.

**Methods:**

We systematically searched medical literature databases (Medline, EMBASE, and CINAHL) and grey literature. Two researchers selected publications reporting methodological approaches to guideline-based QI development. In order to describe and compare methodological approaches used in these publications, we extracted detailed information on common steps of guideline-based QI development (topic selection, guideline selection, extraction of recommendations, QI selection, practice test, and implementation) to predesigned extraction tables.

**Results:**

From 8,697 hits in the database search and several grey literature documents, we selected 48 relevant references. The studies were of heterogeneous type and quality. We found no randomized controlled trial or other studies comparing the ability of different methodological approaches to guideline-based development to generate high-quality QIs. The relevant publications featured a wide variety of methodological approaches to guideline-based QI development, especially regarding guideline selection and extraction of recommendations. Only a few studies reported patient involvement.

**Conclusions:**

Further research is needed to determine which elements of the methodological approaches identified, described, and compared in this review are best suited to constitute a gold standard for guideline-based QI development. For this research, we provide a comprehensive groundwork.

## Background

According to the definition of the Institute of Medicine (1990), quality of care is the "degree to which health services for individuals and populations increase the likelihood of desired health outcomes and are consistent with current professional knowledge" [1,2]. Increasingly, quality indicators (QIs) are employed to assess and improve the quality of care in many healthcare settings [1,3-5]. QIs are measurable items referring to structures, processes, and outcomes of care [6]. They imply a judgment on the quality of care provided. However, the interpretation of such performance assessments can have far-reaching consequences, for instance, in application to pay-for-performance models. Hence, the development of QIs should be based on a systematic approach that ensures transparency and produces high-quality standards [7]. Important attributes of high-quality QIs are their relevance to the selected problem and field of application, their feasibility, and their reliability. They further need to be easily understandable for providers and patients, changeable by behavior, achievable, and measurable with high validity [8,9]. To ensure content and construct validity, QIs need to be evidence based and should have a strong correlation with the actual quality of care provided, respectively [9,10]. The reliability of QIs in regard to their level of measurement error can be assessed by an evaluation of the intra- and inter-observer reliability [11].

State-of-the-art methodological approaches to QI development have been described in several studies [12-15], and a large body of literature exists evaluating their strengths and limitations [13,16,17]. However, to date, no study of which we are aware exists that systematically compares different methodological approaches to QI development with respect to their ability to generate QIs that improve the quality of the particular healthcare aspects they were designed for.

Developing QIs is an expensive and time-consuming process. They are usually specific to certain healthcare settings and, as a result, cannot always be applied to other settings without an adequate adaption process [17]. A time-efficient and resource-saving approach is either to generate QIs from clinical guidelines already available or to couple the process of guideline development with the formulation of appropriate QIs [18,19]. Due to the aim of clinical practice guidelines to improve quality-of-care processes in practices and care institutions, guideline-based QIs predominantly relate to process quality. However, no gold standard exists for guideline-based QI development [10,20,21].

Blozik *et al. *[20] recently conducted a survey among members of the Guideline International Network (G-I-N [Guidelines International Network, Perthshire, Scotland]) that shows that even among working groups specializing in guideline and QI development, a wide variety of methodological approaches are used. A gold standard would help to standardize procedures, foster transparency, and improve efficiency of resources used.

This review aims to identify, describe, and compare methodological approaches to guideline-based QI development. By pooling the available knowledge and appraising strengths and limitations, we intend to provide the groundwork necessary for defining a gold standard for the development of QIs from clinical practice guidelines. To achieve this, we addressed the following research questions:

1. Which methodological approaches to guideline-based development of QIs have been described so far?

2. What are the strengths and limitations of the methodological approaches described regarding their ability to generate high-quality QIs?

3. Do methodological approaches to the development correlate with the quality of QIs they produce?

## Methods

We carried out a systematic literature search across three electronic databases: MEDLINE (US National Library of Medicine, Bethesda, MD, USA), the Excerpta Medica database (Embase [Elsevier B.V., New York, NY, USA]; both via OvidSP^® ^[Ovid Technologies, Inc., New York, NY, USA]) to cover articles in medical journals that are not included in MEDLINE, and the Cumulative Index to Nursing and Allied Health Literature (CINAHL [EBSCO Publishing, Ipswich, MA, USA]) to include articles published in the field of nursing and the allied health professions. The query date of all three databases was April 22, 2010. The search included literature from the earliest records available in the databases up to the search date. Duplicates were eliminated both manually and automatically. To identify articles for review, we linked three search columns using the Boolean operator "and": quality indicators, guidelines, and development. We combined several search terms with the Boolean operator "or" in order to operationalize the search terms (the MEDLINE search algorithm can be found in Additional file 1: Table S1 and was slightly adapted for Embase and CINAHL). We drew several search terms from the controlled vocabularies used for subject indexing in MEDLINE (*i.e*., Medical Subject Headings [MeSH]), Embase (*i.e*., EMTREE), and CINAHL (*i.e*., CINAHL Subject Headings). We searched three databases for ongoing studies (Current Controlled Trials [Springer Science & Business Media, New York, NY, USA], HSRProj [Health Services Research Projects in Progress, US National Library of Medicine, Bethesda, MD, USA], UKCRN-Portfolio [United Kingdom Clinical Research Network, National Institute for Health Research, London, UK] [22]). In addition, we screened the reference lists of all retrieved publications included in the final review. From the relevant literature and the G-I-N database, we derived contact information of institutions and working groups in the field of guideline and QI development. We scanned relevant government and institutional websites in order to obtain web-published documents such as method papers (for details of websites searched, see Additional file 2: Table S2). Finally, we consulted colleagues with a research interest in QI to point out articles not identified during our database, websites, and reference list search.

Two reviewers independently screened all obtained references for eligibility in a three-stage screening process. Discrepancies were solved by consensus. Articles were considered for inclusion if they reported at least one methodological approach to guideline-based QI development and if they were published in English, French, or German. All study and publication types were included.

**Figure 1:**
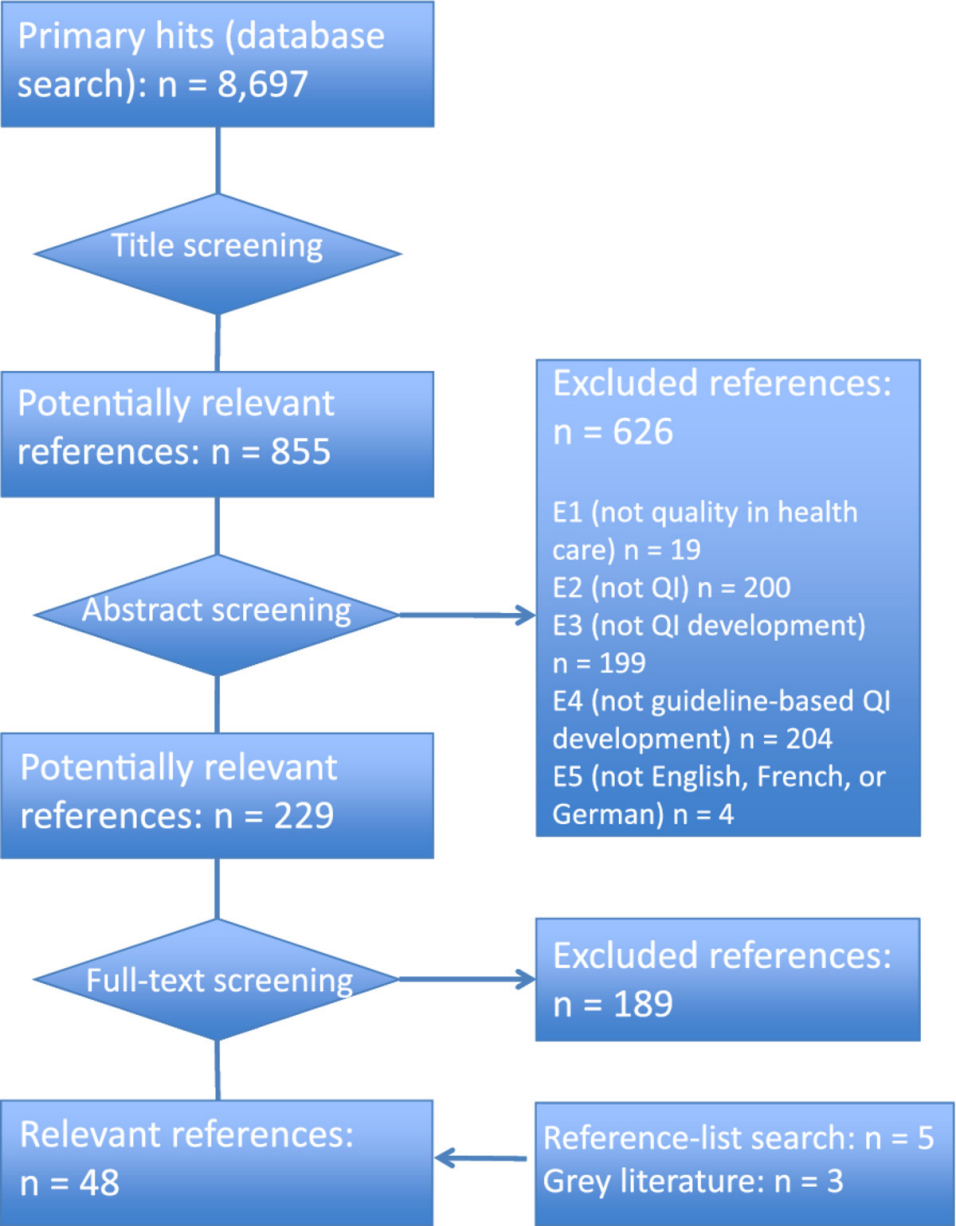
**Flowchart summarizing the screening process**.

The detailed reporting of the individual development steps (see next paragraph) in publications describing methodological approaches to QI development is indispensable for their reconstruction--be it for the purpose of process evaluation (as we did) or in order to apply methodological approaches to QI development in other settings. We therefore excluded studies at the full-text screening stage that did not describe the extraction of recommendations from clinical guidelines in detail, as this was the process of particular interest to this review. Details of the selection process, including exclusion criteria at the abstract-screening stage, are summarized in Figure 1.

**Figure 2:**
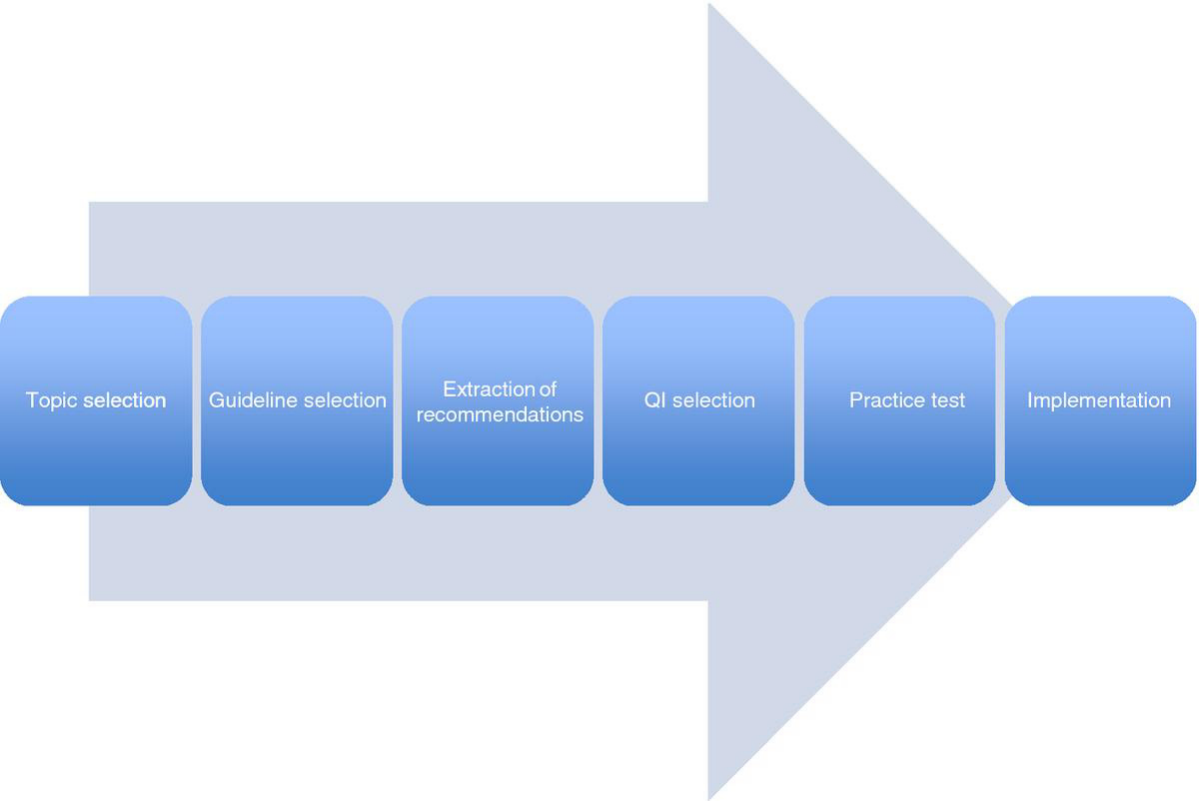
**Overview of the process of guideline-based QI development**.

Two researchers independently extracted data from the relevant literature to a predesigned data extraction form (see Additional file 3: Table S3); discrepancies were solved by consensus. In order to describe and to compare methodological approaches to guideline-based QI development, we developed an *a priori *framework of the QI development process. For this purpose, we identified six steps that most methodological approaches to guideline-based QI development have in common with regard to function and succession but that differ in their design from one methodological approach to another. Through a preliminary search and analysis of a select number of key publications, we identified six development steps: (1) topic selection, (2) guideline selection, (3) extraction of recommendations, (4) QI selection, (5) practice test, and (6) implementation (see Figure 2). The data extraction form was specifically designed to include (a) information about the methodological approach to these six development steps and (b) items necessary to perform a quality assessment of the relevant studies. For steps 1 to 4, we extracted information about how and by whom the specific development step was conducted, such as selection criteria for topics, guidelines, and recommendations, as well as participants. The two development steps specific to guideline-based QI development (compared to QI development from other sources) were investigated in more detail, namely, guideline selection and extraction of recommendations. In addition to the above-mentioned selection criteria, we collected information about the selected guidelines (Was some sort of quality assessment conducted? Were all selected guidelines listed in the publication?), as well as the extracted recommendations (Were they reported at all? If yes, were the source guideline and the underlying level of evidence made transparent?). For an overview of all selected information on guideline selection and extraction of recommendations, see Table 1.

**Table 1:** Information extracted relating to guideline selection and extraction of recommendations

Guideline selection	Extraction of recommendations
Were QIs developed from • one guideline, • more than one guideline, or • guidelines and other sources?	Were • all recommendations or • a selection of recommendations extracted?

Which criteria for guideline selection were reported?	If not all recommendations were extracted, which criteria were reported for their selection?

Did the authors report a critical appraisal of selected guidelines?	Who did extraction recommendations?

Were the selected guidelines listed in the publication?	Which criteria were reported for the selection of persons involved in recommendation extraction?

Who selected the guidelines?	Were the extracted recommendations reported in the publication or additional files available to the reader?

Which criteria were reported for the selection of persons involved in guideline selection?	Did the authors report sources/levels of evidence of the extracted recommendations?

QI = quality indicator

Due to the wide variety of study and publication types and the overlap of the quality assessment and the assessment of methodological approaches, we limited the quality assessment to items covering funding information, the reporting of study and publication type, and the reporting of duration and time frame of the study.

Following data extraction and identification of the methodological approaches to each of the above-listed development steps, we focused on analyzing the similarities and differences among the identified methodological approaches. The results are presented following further elaboration of the six development steps introduced above. We discuss our results in context of the current literature in the Discussion section.

## Results

### Search findings and literature selection

We identified a total of 8,697 potentially relevant articles, of which 8,468 were excluded based on their titles or abstracts (see Figure 1 for details regarding the screening process). No additional articles were identified through expert consultation. We conducted full-text reviews of the remaining 229 articles and an additional eight articles identified in reference lists and in the grey literature. The final review included 48 articles.

Of the 48 articles in the final review, 10 papers described methodological approaches to guideline-based QI development in general (referred to as "method papers") [1,7,23-30], and 32 articles [31-62] addressed the guideline-based QI development for a certain clinical topic (referred to as "topic papers"). An additional six papers [10,19,63-66] comprised a detailed description of a method as well as its application for a certain clinical topic (referred to as "method + topic papers"). None of the selected publications was a controlled study comparing one development method to another. All journal articles were published in English; two of the method papers published via institutional websites [25,26] were written in German.

In not disclosing the funding source and time frame of the study and in not explicitly reporting the study type, many of the publications did not meet our basic quality-assessment criteria (for details, see Table 2).

**Table 2:** Characteristics of included references: General characteristics and quality assessment

*General characteristics*				*Quality assessment*		
***Reference***	***Institution***	***Topic***	***Setting***	***Study/publication type mentioned***	***Study duration mentioned***	***Funding***

**Method papers**						

ÄZQ (2009)	ÄZQ (Berlin, DE)	-	-	No	n/a	Unclear

AHCPR (1995)	AHRQ (Rockville, MD, US)	-	-	No	n/a	Unclear

AHRQ (1995)	AHRQ (Rockville, MD, US)	-	-	Yes - report	n/a	Combined public/private

AQUA (2010)	AQUA (Göttingen, DE)	-	-	Yes - method paper	n/a	Unclear

Baker and Fraser (1995)	Eli Lilly National Clinical Audit Centre (Leicester, UK)	-	-	Yes - review	n/a	Unclear

Bergman (1999)	Dept. of Pediatrics, Stanford School of Medicine (Palo Alto, CA, US)	-	-	No	n/a	Unclear

Califf *et al. *(2002)	DCRI (Durham, NC, US)	-	-	Yes - state-of-the-art paper	n/a	Public

Campbell *et al. *(2002)	NPCRDC (Manchester, UK)	-	-	Yes - review	n/a	Unclear

Graham *et al. *(2009)	Immpact (Aberdeen, UK)	-	-	Yes - review	n/a	Public

Spertus *et al. *(2005)	AHA (Dallas, TX, US)	-	-	No	n/a	Public

**Topic papers**						

Bonow *et al. *(2005)	AHA (Dallas, TX, US)	Heart failure	Hospital/outpatient care	Yes - report	No	Public

Burge *et al. *(2007)	CCORT (Toronto, CA)	Heart failure	Primary care	No	No	Public

Campbell *et al. *(1999)	NPCRDC (Manchester, UK)	CHD, Type 2 Diabetes, Asthma	Primary care	Yes - original article	No	Unclear

Desch *et al. *(2008)	RPCI (Buffalo, NY, US)	Breast cancer	Hospital care	Yes - special article	No	Public

Draskovic *et al. *(2008)	IQ healthcare (Nijmegen, NL)	Dementia	Hospital care	No	No	Public

Estes *et al. *(2008)	AHA (Dallas, TX, US)	Atrial fibrillation	Outpatient care	Yes - report	No	Public

Forbes *et al. *(1997)	KU School of Nursing (Kansas City, MO, US)	Stroke	Rehabilitation	No	No	Public

Giesen *et al. *(2007)	IQ healthcare (Nijmegen, NL)	Prescribing and referral	Emergency primary care	No	No	Unclear

Hadorn *et al. *(1996)	RAND (Santa Monica, CA, US)	Heart failure	Primary care	Yes - article	No	Combined public/private

Hardy and Hadley (1995)	CCQE (Washington, DC, US)	Pain	All	No	No	Unclear

Hermanides *et al. *(2008)	IQ healthcare (Nijmegen, NL)	Urinary tract infection	Hospital care	Yes - major article	No	Unclear

Hermens *et al. *(2006)	IQ healthcare (Nijmegen, NL)	Lung cancer	Hospital care	Yes - article	No	Public

James *et al. *(1997)	Office of Rural Health (Buffalo, NY, US)	Heart failure	Primary care	Yes - paper	No	Public

Kongnyuy and van den Broek (2008)	LSTM (Liverpool, UK)	Perinatal care	Hospital care	Yes - research article	No	Combined public/private

Krumholz *et al. *(2006)	AHA (Dallas, TX, US)	Myocardial infarction	Hospital care	Yes - report	No	Public

Lee *et al. *(2003)	CCORT (Toronto, CA)	Heart failure	Hospital/outpatient care	Yes - clinical study	No	Public

MacLean *et al. *(2004)	RAND (Santa Monica, CA, US)	Rheumatoid arthritis	All	Yes - original article	No	Unclear

Martirosyan *et al. *(2008)	IQ healthcare (Nijmegen, NL)	Type 2 Diabetes	Primary care	Yes - original research	No	Public

Mourad *et al. *(2007)	IQ healthcare (Nijmegen, NL)	Subfertility care	All	No	No	Public

Nijkrake *et al. *(2009)	IQ healthcare (Nijmegen, NL)	Parkinson's disease	Physiotherapy	No	No	Public

Ouwens *et al. *(2007)	IQ healthcare (Nijmegen, NL)	Head and neck cancer	Cross-sectoral care	Yes - original article	No	Public

Ouwens *et al. *(2010)	IQ healthcare (Nijmegen, NL)	Patient-centered care	All	Yes - original article	No	Unclear

Radtke *et al. *(2009)	CVderm (Hamburg, DE)	Psoriasis vulgaris	All	Yes - original paper	No	Unclear

Redberg *et al. *(2009)	AHA (Dallas, TX, US)	Cardiovascular prevention	All	Yes - report	No	Public

Schouten *et al. *(2005)	IQ healthcare (Nijmegen, NL)	Pneumonia	Hospital care	yes - major article	No	unclear

Sugarman *et al. *(2003)	Qualis Health (Seattle, WA, US)	Dialysis	All	Yes - special article	Yes	Public

Thomas *et al. *(2007)	AHA (Dallas, TX, US)	Cardiovascular diseases	Rehabilitation	No	No	Public

Tu *et al. *(2008)	CCORT (Toronto, CA)	Myocardial infarction	Hospital care	Yes - review	No	Public

van den Boogaard *et al. *(2010)	IQ healthcare (Nijmegen, NL)	Miscarriage	All	Yes - article	No	Public

van Hulst *et al. *(2009)	IQ healthcare (Nijmegen, NL)	Rheumatoid arthritis	All	Yes - extended report	No	Unclear

Wang *et al. *(2006)	RAND (Santa Monica, CA, US)	Preterm birth	Outpatient care	Yes - article	No	Public

Yazdany *et al. *(2009)	UCSF (San Francisco, CA, US)	Lupus erythematodes	All	Yes - original article	No	Unclear

**Method + topic papers**						

Advani *et al. *(2003)	BMIR (Stanford, CA, US)	Hypertension	All	No	No	Public

Duffy *et al. *(2005)	APIRE (Arlington, VA, US)	Bipolar disorder	Outpatient care	No	No	Unclear

Golden *et al. *(2008)	UAMS (Little Rock, US)	Bipolar disorder	Outpatient care	No	No	Public

Hutchinson *et al. *(2003)	ScHARR (Sheffield, UK)	CHD	Primary care	Yes - original paper	Yes	Combined public/private

LaClair *et al. *(2001)	VA Medical Center (Kansas City, MO, US)	Stroke	Rehabilitation	No	No	Public

Wollersheim *et al. *(2007)	IQ healthcare (Nijmegen, NL)	Oncology, Type 2 Diabetes, pneumonia	All	Yes - review article	No	Unclear

ÄZQ = Ärztliches Zentrum für Qualität in der Medizin (Agency for Quality in Medicine); AHCPR = Agency for Healthcare Policy and Research; AHRQ = Agency for Healthcare Research and Quality; AQUA-Institute = Institute for Applied Improvement and Research in Health Care; DCRI = Duke Clinical Research Institute; NPCRDC = National Primary Care Research and Development Council; Immpact = Initiative for Maternal Mortality Programme Assessment; CCORT = Canadian Cardiovascular Outcomes Research Team; CHD = coronary heart disease; RPCI = Roswell Park Cancer Institute; AHA = American Heart Association; CCQE = Center for Clinical Quality Evaluation; LSTM = Liverpool School of Tropical Medicine; CVderm = Competenzzentrum Versorgungsforschung in der Dermatologie (Institute for Health Services Research in Dermatology); UCSF = University of California, San Francisco; BMIR = Center for Biomedical Informatics Research; APIRE = American Psychiatric Institute for Research and Education; UAMS = University of Arkansas for Medical Sciences; ScHARR = School of Health and Related Research.

The identified relevant studies originate from many different institutions and working groups, only a few of which have published more than one relevant study on guideline-based QI development (*e.g*., the Dutch IQ healthcare [University of Radbound, Nijmegen, The Netherlands]).

**Figure 3:**
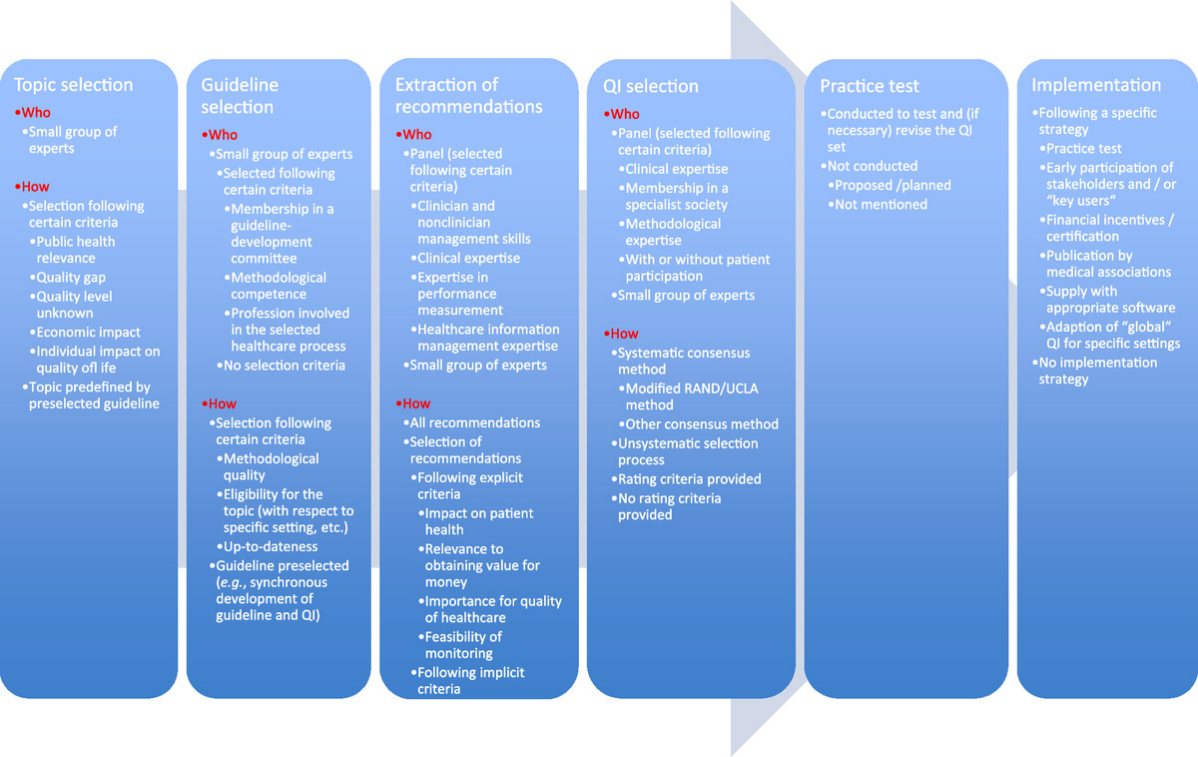
**Methodological variability of guideline-based QI development**.

Tables 2, 3, and 4 provide an overview of the characteristics of all included publications. Figure 3 provides a comprehensive overview of all methodological approaches identified.

**Table 3:** Characteristics of included references: Methodological approaches to topic/guideline selection and extraction of recommendations

	Topic/guideline selection							Extraction of recommendations		
***Reference***	***Criteria for selection of topic***	***Development of QI from...***	***Criteria for selection of participants***	***Criteria for selection of guidelines***	***Participants listed^a^***	***Critical appraisal***	***Guidelines listed^a^***	***Extraction of all/a selection of recommendations***	***Criteria for recommendation selection^b^***	***Potential indicators listed^a^***

**Method papers**										

ÄZQ (2009)	No	One guideline	No	No	-	No	-	Unclear	-	-

AHCPR (1995)	No	One guideline	Yes Profession involved in the selected healthcare process, methodological competence	Yes Methodological quality	-	Yes Not detailed	-	Selection	Yes Impact on patient outcome	-

AHRQ (1995)	Yes Regulatory requirements, quality gap, guideline adherence unknown	More than one guideline	No	Yes Methodological quality	-	Yes Not detailed	-	Selection	Yes Impact on patient outcome and relevance to obtaining value for money	-

AQUA (2010)	Yes Public health relevance, sound evidence base, feasibility	Guidelines and other sources	No	Yes Methodological quality	-	Yes AGREE Instrument	-	All	-	-

Baker and Fraser (1995)	No	Not specified (method paper)	No	No	-	Yes Not detailed	-	Unclear	-	-

Bergman (1999)	Yes Sound evidence base	Not specified (method paper)	No	No	-	Yes Not detailed	-	Unclear	.	-

Califf *et al. *(2002)	No	One guideline	No	No	-	Yes Not detailed	-	Selection	Yes Level of evidence	-

Campbell *et al. *(2002)	No	Not specified (method paper)	No	No	-	No	-	Unclear	-	-

Graham *et al. *(2009)	Yes Quality gap	Guidelines and other sources	No	No	-	No	-	Unclear	-	-

Spertus *et al. *(2005)	No	Not specified (method paper)	No	Yes Strength of evidence, clinical relevance, magnitude of relationship between performance and outcome	-	Yes Not detailed	-	Selection	Yes Level of evidence, impact on patient outcome	-

**Topic papers**										

Bonow *et al. *(2005)	Yes Public health relevance, quality gap, costs	More than one guideline	No	No	Yes	Yes Not detailed	Yes	Selection	Yes Grade of recommendation, relevance for the topic	No

Burge *et al. *(2007)	Yes Public health relevance, quality gap	Unclear	No	No	Yes	No	No	Selection	Yes Potential for improvement, meaningful, valid, reliable, adjustable, feasible	No

Campbell *et al. *(1999)	Yes Public health relevance, substantial amount of workload in general practice	Guidelines and other sources	No	No	No	No	Yes	Unclear	-	No

Desch *et al. *(2008)	No	Guidelines and other sources	Yes Profession involved in the selected healthcare process	No	Unclear	No	Yes	Selection	Yes Impact on patient outcome, potential for improvement, feasibility of data collection	No

Draskovic *et al. *(2008)	Yes Variance in quality of care between providers	One guideline	No	No	No	No	Yes	Unclear	-	No

Estes *et al. *(2008)	Yes Public health relevance and costs	Guidelines and other sources	No	No	Yes	Yes Not detailed	Yes	Selection	Yes Grade of recommendation, relevance for the topic	No

Forbes *et al. *(1997)	Yes Public health relevance, individual impact on quality of life	One guideline	No	No	No	No	Yes	All	-	No

Giesen *et al. *(2007)	Yes Quality of care unknown	Guidelines and other sources	No	Yes Applicability to the setting, clinical relevance	Yes	Yes AGREE instrument	Yes	Selection	Yes Relevance for the selected topic	No

Hadorn *et al. *(1996)	Yes Public health relevance, individual quality-of-life impact, costs	One guideline	No	No	Yes	No	Yes	All	-	Yes

Hardy and Hadley (1995)	No	One guideline	No	Unclear	No	No	Yes	Unclear	-	No

Hermanides *et al. *(2008)	Yes Public health relevance, quality gap	One guideline	No	No	No	No	Yes	Selection	No	Yes

Hermens *et al. *(2006)	Yes Quality of care unknown, guideline adherence unclear	One guideline	No	No	No	No	Yes	All	-	No

James *et al. *(1997)	Yes Public health relevance, costs, quality gap	One guideline	No	No	No	Yes Not detailed	Yes	All	-	No

Kongnyuy and van den Broek (2008)	No	Guidelines and other sources	No	No	No	No	Yes	Unclear	-	No

Krumholz *et al. *(2006)	Yes Public health relevance, quality gap	More than one guideline	No	No	Yes	Yes Not detailed	Yes	Selection	Yes Grade of recommendation	No

Lee *et al. *(2003)	No	Guidelines and other sources	No	No	No	No	Yes	Unclear	-	No

Maclean *et al. *(2004)	Yes Public health relevance	Guidelines and other sources	No	No	No	Unclear	Yes	Selection	Yes Impact on patient outcome, grade of recommendation	No

Martirosyan *et al. *(2008)	Yes Public health relevance, quality of care unknown	More than one guideline	No	No	No	No	Yes	Selection	Yes Measurability	Yes

Mourad *et al. *(2007)	Yes Public health relevance, quality gap	More than one guideline	No	Yes Methodological quality	No	No	Yes	All	-	No

Nijkrake *et al. *(2009)	Yes Public health relevance and complexity of the topic	One guideline	No	No	No	No	Yes	Selection	Yes Acceptability, measurability	No

Ouwens *et al. *(2007)	Yes Complexity of the process of care	Guidelines and other sources	No	No	No	No	Yes	Selection	Yes Impact on patient outcome	No

Ouwens *et al. *(2010)	Yes Individual impact on quality of life, quality gap	Guidelines and other sources	No	Yes Applicability to the setting	No	No	Yes	All	-	No

Radtke *et al. *(2009)	No	Guidelines and other sources	No	No	No	Yes Not detailed	Yes	Unclear	-	No

Redberg *et al. *(2009)	Yes Public health relevance, costs, quality gap	One guideline	No	No	No	No	Yes	Selection	Unclear	No

Schouten *et al. *(2005)	Yes Quality gap	Guidelines and other sources	No	No	No	No	Yes	Selection	No	Yes

Sugarman *et al. *(2003)	Yes Quality of care unknown, regulatory requirements	One guideline	No	No	No	No	Yes	Unclear	-	No

Thomas *et al. *(2007)	Yes Underutilization, quality of care unknown	Guidelines and other sources	No	No	Yes	Yes Not detailed	Yes	Selection	Yes Grade of recommendation, level of evidence	No

Tu *et al. *(2008)	Yes Quality gap	Guidelines and other sources	No	No	Yes	No	Yes	Selection	Yes Meaningful, valid and reliable, feasible, accountable for patient variability, potential for improvement,	No

van den Boogaard *et al. *(2010)	Yes Quality gap	One guideline	No	Yes Most recently revised guideline available	No	No	Yes	All	-	No

van Hulst *et al. *(2009)	No	Guidelines and other sources	No	No	No	No	Yes	Selection	Yes Grade of recommendations	No

Wang *et al. *(2006)	Yes Public health relevance, complex process of care, quality gap	Guidelines and other sources	No	No	Yes	No	No	Selection	Yes Impact on patient outcome, level of evidence, potential for improvement, feasibility of data collection	No

Yazdany *et al. *(2009)	Yes Quality of care unknown	Guidelines and other sources	No	Yes Methodological quality	Yes	Unclear	No	Selection	Yes Eligible population, process of care performed by healthcare providers, impact on patient outcome	No

**Method + topic papers**										

Advani *et al. *(2003)	No	One guideline	No	No	No	No	Yes	Unclear	-	No

Duffy *et al. *(2005)	Yes Individual impact on quality of life, quality gap	More than one guideline	No	No	No	No	Yes	Selection	Yes Level of evidence, impact on patient outcome, breadth of available treatment recommendations, clinical utility and appropriateness, proportion of patients for whom the recommendation is likely to be relevant	No

Golden *et al. *(2008)	Yes Public health relevance, costs, quality gap	Guidelines and other sources	Yes Profession involved in the selected health care process	No	No	No	No	Selection	Yes Level of evidence	No

Hutchinson *et al. *(2003)	No	More than one guideline	No	Yes Evidence based	No	Yes Suitable for primary care, agency responsible for development clearly identifiable, objectives clearly defined, independent review prior to publication, information regarding evidence adequate and explicit, link between major recommendations and underlying evidence	Yes	Selection	Unclear	No

Laclair *et al. *(2001)	No	One guideline	No	No	Yes	No	Yes	All	-	No

Wollersheim *et al. *(2007)	Yes Quality gap, public health relevance, sound evidence base	Guidelines and other sources	Yes Membership in a guideline-development committee, methodological competence, profession involved in the selected healthcare process	No	No	No	Yes	Unclear	-	No

QI = quality indicator; ÄZQ = Ärztliches Zentrum für Qualität in der Medizin (Agency for Quality in Medicine); AHCPR = Agency for Healthcare Policy and Research; AHRQ = Agency for Healthcare Research and Quality; AQUA-Institute = Institute for Applied Improvement and Research in Health Care; AGREE = Appraisal of Guidelines for Research and Evaluation in Europe.

^a^Does not apply to method papers; ^b^does apply if not all recommendations are extracted.

**Table 4:** Characteristics of included references: Methodological approaches to QI selection, practice test, and implementation

	QI selection							Additional QI development elements		
***Reference***	***Panel method***	***Criteria for panel members***	***Panel members listed^a^***	***Selected indicators listed^a^***	***Sources transparent^1^***	***LoE^b^***	***Rating criteria***	***Practice test***	***Implementation strategy***	***Patient participation***

**Method papers**										

ÄZQ (2009)	Unclear	Unclear	-	-	-	Yes	Yes Importance for the healthcare system, clarity, improvability, risk for adverse effect, evidence base, grade of recommendation	Proposed	No	No

AHCPR (1995)	No	No panel method	-	-	-	No	Unclear	Not mentioned	No	No

AHRQ (1995)	No	No panel method	-	-	-	No	No	Included	Yes Development of data collection software, audit and feedback	No

AQUA (2010)	Modified RAND/UCLA	Yes Clinical expertise, methodological expertise	-	-	-	Yes	Yes Relevance, clarity, feasibility	Included	Yes Development/upgrading of data collection software	QI selection

Baker and Fraser (1995)	No	No panel method	-	-	-	No	Unclear	Not mentioned	Yes Local development, ownership	No

Bergman (1999)	No	No panel method	-	-	-	Yes	Unclear	Proposed	Yes Involving key stakeholders	No

Califf *et al. *(2002)	No	No panel method	-	-	-	Yes	Unclear	Not mentioned	Yes Education and feedback	No

Campbell *et al. *(2002)	Other	Unclear	-	-	-	No	Unclear	Not mentioned	No	No

Graham *et al. *(2009)	Other	No	-	-	-	No	Yes Grade of recommendation, level of evidence, measurability, improvability	Included	Yes Audit and feedback	No

Spertus *et al. *(2005)	No	No panel method	-	-	-	No	Yes Useful in improving patient outcomes, measure design, measure implementation, overall assessment	Not mentioned	No	No

**Topic papers**										

Bonow *et al. *(2005)	Other	No	Yes	Yes	Yes	Yes	Yes Useful in improving patient outcomes, measure design, measure implementation, overall assessment	Not mentioned	Yes Defining challenges to implementation for each QI	No

Burge *et al. *(2007)	Modified RAND/UCLA	Yes Members of specialist societies	Yes	Yes	In part	No	No	Proposed	No	No

Campbell *et al. *(1999)	Modified RAND/UCLA	Yes Clinical expertise, members of specialist societies	No	Yes	In part	Yes	No	Not mentioned	Yes	No

Desch *et al. *(2008)	Other	Yes Members of specialist societies, methodological expertise	Yes	Yes	Yes	No	No	Not mentioned	Yes Integration in nationwide quality-improvement programs	No

Draskovic *et al. *(2008)	Modified RAND/UCLA	Yes Clinical expertise	No	Yes	Yes	No	Yes Face validity	Included	Yes Including the informal caregivers' perspective	No

Estes *et al. *(2008)	Other	No	Yes	Yes	Yes	Yes	Yes Useful to improve patient outcomes, measure design, measure implementation, overall assessment	Not mentioned	Yes Defining challenges to implementation for each QI	No

Forbes *et al. *(1997)	No	No panel method	No panel method	Yes	Yes	No	No	Included	Yes Pilot testing	No

Giesen *et al. *(2007)	Other	Unclear	No	Yes	In part	No	Yes Relevance, utility for evaluation of care	Included	No	No

Hadorn *et al. *(1996)	Unclear	No	No	Yes	In part	No	Unclear	Not mentioned	No	No

Hardy and Hadley (1995)	Unclear	Unclear	No	No	Yes	No	No	Not mentioned	No	No

Hermanides *et al. *(2008)	Other	Yes Clinical expertise	Yes	Yes	Yes	Yes	Yes Appropriateness	Included	No	No

Hermens *et al. *(2006)	Modified RAND/UCLA	Yes Clinical expertise	Yes	Yes	Yes	No	Yes Professional quality, organisational quality, patient-oriented quality	Included	Yes Practice test	QI selection

James *et al. *(1997)	Other	Yes Clinical expertise	No	Yes	Yes	Yes	Yes Educational appropriateness, clinical importance, measurement feasibility	Not mentioned	No	No

Kongnyuy and van den Broek (2008)	Other	Yes Clinical expertise, laypersons	No	Yes	In part	No	No	Planned	Yes Involving all grades of health professionals during the whole development process	QI selection

Krumholz *et al. *(2006)	Other	Yes Clinical expertise, methodological expertise members of specialist societies	Yes	Yes	Yes	Yes	Yes Useful in improving patient outcomes, measure design, measure implementation, overall assessment	Not mentioned	Yes Defining challenges to implementation for each QI	No

Lee *et al. *(2003)	Other	Yes Clinical expertise	Yes	Yes	In part	No	Yes Meaningfulness, usefulness, potential for improvement, impact on patient outcomes, feasibility of data collection	Not mentioned	No	No

Maclean *et al. *(2004)	Modified RAND/UCLA	Yes linical expertise, methodological expertise members of specialist societies	Yes	Yes	No	Yes	Unclear	Not mentioned	No	No

Martirosyan *et al. *(2008)	Modified RAND/UCLA	Yes Clinical expertise, methodological expertise members of specialist societies	No	Yes	In part	No	Unclear	Included	No	No

Mourad *et al. *(2007)	Modified RAND/UCLA	Yes Clinical expertise, methodological expertise	No	Yes	Yes	Yes	Unclear	Proposed	Yes Practice test	No

Nijkrake *et al. *(2009)	Other	Yes Clinical expertise, methodological expertise	No	No	Yes	Yes	Yes Relevance (effectiveness, efficiency, acceptability, measurability)	Included	Yes Training in the correct use of the respective guideline	No

Ouwens *et al. *(2007)	Modified RAND/UCLA	Yes Clinical expertise	No	Yes	In part	No	Yes Clinically relevant to patients' health benefits and/or to the continuity and coordination of care	Included	Yes Practice test	QI selection

Ouwens *et al. *(2010)	Other	Yes Patient representatives	No	Yes	In part	No	Unclear	Included	Yes Patient participation	QI selection

Radtke *et al. *(2009)	Other	Yes Clinical expertise, methodological expertise, patients	No	Yes	In part	No	Yes Inclusion in the research literature, measurable under routine conditions, inclusion in a certain high-quality guideline, reproducibility, validity, clinical relevance, sensitivity to change	Included	No	No

Redberg *et al. *(2009)	Other	Yes Clinical expertise, methodological expertise membership in specialist societies	Yes	Yes	Yes	Yes	Yes Useful in improving patient outcomes, measure design, measure implementation, overall assessment	Not mentioned	No	No

Schouten *et al. *(2005)	Modified RAND/UCLA	Yes Clinical expertise, methodological expertise	No	Yes	Yes	Yes	Yes Clinical relevance to the patient's health benefit, relevance to reducing antimicrobial resistance, relevance to cost effectiveness	Included	No	No

Sugarman *et al. *(2003)	Other	Yes Clinical expertise, membership in specialist societies	No	No	Yes	Yes	Yes Clinical importance, feasibility of measurement, level of evidence	Included	No	No

Thomas *et al. *(2007)	Unclear	Yes Clinical expertise, methodological expertise, membership in specialist societies	Yes	Yes	Yes	Yes	Yes Evidence based, interpretable, actionable, clinically meaningful, valid, reliable, feasible	Not mentioned	Yes Defining challenges to implementation for each QI	No

Tu *et al. *(2008)	Other	Yes Clinical expertise, methodological expertise, membership in specialist societies	Yes	Yes	In part	No	Yes Usefulness in improving patient outcomes, feasibility of data collection, reliability, validity	Not mentioned	Yes Pay for performance, collaboration with national and local initiatives, use of standard tools, presentation at scientific meetings, availability online	No

van den Boogaard *et al. *(2010)	Modified RAND/UCLA	Yes Clinical expertise	Yes	Yes	Yes	Yes	Yes Health gain, overall efficacy	Proposed	No	No

van Hulst *et al. *(2009)	Modified RAND/UCLA	Yes Clinical expertise, methodological expertise	No	Yes	In part	Yes	No	Not mentioned	Yes Using understandable and measurable QIs	No

Wang *et al. *(2006)	Other	Yes Membership in specialist societies	No	Yes	In part	Yes	Yes Validity, feasibility	Not mentioned	No	No

Yazdany *et al. *(2009)	Modified RAND/UCLA	Yes Clinical expertise, methodological expertise	Yes	Yes	No	Yes	Yes Evidence base, validity, feasibility	Proposed	Yes Assess the technical characteristics of developed QIs	No

**Method + topic papers**										

Advani *et al. *(2003)	No	No panel method	No panel method	No	Yes	No	No	Included	No	No

Duffy *et al. *(2005)	Unclear	Unclear	No	Yes	Yes	Yes	Unclear	Planned	Yes Integration in health plan performance measurement, quality monitoring and accreditation programs, integration of needed data elements in medical information systems	No

Golden *et al. *(2008)	Modified RAND/UCLA	Yes Clinical expertise, methodological expertise, laypersons	No	No	In part	No	Yes Meaningfulness, quality gap, improvability, feasibility of data collection	Included	Yes Transparency during the development process, providing the data collection tool, submission to a national performance measurement program	QI selection

Hutchinson *et al. *(2003)	Other	Yes Clinical expertise	No	Yes	In part	Yes	No	Not mentioned	No	No

Laclair *et al. *(2001)	Other	Yes Clinical expertise, methodological expertise	No	No	Yes	Yes	No	Included	No	No

Wollersheim *et al. *(2007)	Modified RAND/UCLA	Yes Clinical expertise, methodological expertise	No	Yes	In part	Unclear	No	Included	Yes Periodic audits	No

QI = quality indicator; ÄZQ = Ärztliches Zentrum für Qualität in der Medizin (Agency for Quality in Medicine); AHCPR = Agency for Healthcare Policy and Research; AHRQ = Agency for Healthcare Research and Quality; AQUA-Institute = Institute for Applied Improvement and Research in Health Care.

^a^Does not apply to method papers; ^b^LoE = Level of evidence (reported for underlying recommendations of the QI).

Unless indicated otherwise, numbers of studies referred to in the following paragraphs always relate to all 48 studies of the final review pool.

### Topic selection

Criteria for the selection of a clinical topic for QI development were detailed in 33 publications. The most frequently reported criteria were

• the public health relevance of a topic (mentioned in 18 publications),

• the existence of a gap between potential and actually achieved quality of healthcare (mentioned in 16 publications).

Other reported criteria were uncertainty about the quality of care provided for a specific healthcare setting (mentioned in six publications), the economical impact of a specific healthcare problem (mentioned in six publications), and the individual impact on the quality of life (mentioned in four publications).

### Guideline selection

In 16 studies, QIs were developed from a single guideline, whereas in seven studies more than one guideline was used to derive QIs. Twenty studies detailed other sources, such as existing QI databases, in addition to clinical guidelines.

Only eight of the authors who developed QIs from more than one source provided a transparent description of the respective sources of final QIs.

Criteria for the selection of guidelines from which the QIs were derived were reported in 10 publications. Reported criteria were

• the methodological quality,

• the up-to-dateness,

• the eligibility of a guideline for the selected topic (*e.g*., with regard to the specific setting).

In 15 publications a critical appraisal of the used guidelines was reported based on the Appraisal of Guidelines Research and Evaluation in Europe (AGREE) instrument [67] or similar quality criteria.

Whilst participants in guideline selection are often mentioned, at least indirectly, for instance by being referred to as "the authors", criteria for their selection were reported in only four publications. These selection criteria were

• member of a guideline development committee,

• having methodological competence,

• belonging to a profession involved in the selected healthcare process.

### Extraction of recommendations

Nine studies extracted all recommendations from selected guidelines. In 25 studies, recommendations were selected during the extraction process and not all recommendations were extracted as potential QIs. Criteria for this selection were reported in 21 of the 25 studies. Criteria for the preselection at the stage of recommendation extraction mentioned by the Agency for Healthcare Research and Quality (AHRQ) are

• the size of the impact on patient health (the AHRQ considers the impact great when an issue affects a few patients severely or affects many patients),

• the relevance to obtaining value for money.

Other criteria for the preselection formulated by Hadorn *et al. *[39] are

• the importance to quality of healthcare provided,

• the feasibility of monitoring.

Other frequently reported criteria were the level of evidence, the grade of recommendation, and measurability.

Levels of evidence and grades of recommendation of the recommendations potential QIs were developed from were reported in 24 studies. Only four studies reported criteria for the selection of persons who extracted potential QIs from guidelines. They were similar to those for persons involved in guideline selection (see above); both tasks were usually carried out by the same group of people.

The AHRQ [24] provides a detailed description of the extraction process, including specifications of participants' necessary skills, as well as criteria for the selection of recommendations to be extracted.

Four requirements for persons involved in the extraction of potential QIs from guidelines postulated by the AHRQ are

• clinician and nonclinician management skills,

• clinical expertise,

• technical expertise in performance measurement,

• healthcare information management expertise.

Another prerequisite for a valid extraction process mentioned in several of the relevant studies requires that the extraction be performed by at least two researchers independently [25,37-39].

### QI selection

In 35 studies, a consensus method was used to augment the evidence from literature with expert and layperson opinion by letting a panel rate and select a set of final QIs from a set of potential QIs. In 15 of these 35 publications this method was described as the "modified RAND/UCLA method," named after the RAND/UCLA (University of California, Los Angeles) appropriateness method [68].

Whereas only a few studies named the individual members of the panels, criteria for their selection (*e.g*., clinical expertise, methodological expertise, membership in a specialist society) were reported in 31 of 35 studies. Only 25 of 35 studies provided rating criteria for the panel process. Among the frequently named criteria were the usefulness of QIs for improving patient outcomes, their relevance, and the feasibility of monitoring.

Participation of patients in the development process was reported in six studies. In all of these studies, patients participated in the panels. No study reported patient participation during guideline selection and the extraction of recommendations.

### Practice test

Only 19 studies reported the conduct of a QI practice test. In two studies, the practice test was conducted after the development process was completed. In 21 studies, a practice test was not mentioned at all.

### Implementation

An implementation strategy for guideline-based QIs was reported in 26 studies. Among the reported activities were the instruction of key persons ("early adopters") as multipliers, the participation of end users in the development process, the publication of developed QIs by medical associations, supplying the appropriate software, and the adaptation of "global" QIs to more specific settings. Financial incentives and certification were also used to support implementation.

## Discussion

### Topic selection

Authors tended to describe the process of topic selection in insufficient detail. Mostly, selection criteria merely reflected the aims of the application of QIs in general: to measure and improve quality in areas of healthcare where the actual quality of care is either suboptimal or unknown.

### Guideline selection

The selected literature describes two different approaches to guideline selection. The first approach identified in the reviewed literature is to develop QIs based on one or only a few preselected guidelines, often with the aim of supporting or evaluating guideline implementation. In certain contexts, such as specific settings in small healthcare systems, only one guideline may be available for QI development. In these cases, guideline-selection processes are of no or only minor relevance, and the number of recommendations to be translated into potential QIs is proportionately low.

The second approach is to select a clinical topic and, subsequently, to obtain suitable, topic-specific guidelines as a basis for the development of QIs from guideline recommendations. In this case, expert opinion and existing QI sets are sometimes used as alternative sources for QIs. In comparison to the first approach, this approach provides a broader basis for the subsequent development of QIs, bears the potential to produce a balanced set of QIs, carries a reduced risk of selection bias, and increases content validity.

Many studies do not describe their guideline-selection criteria in sufficient detail and lack critical appraisal of their selected guidelines, both of which may compromise content validity and hence the quality of resulting QI sets. We argue that high-quality QIs can only be derived from high-quality guidelines. To ensure QIs originate from a sound foundation, development committees should (a) conduct a systematic search for relevant guidelines in national and international guideline databases as well as conventional literature databases and (b) conduct a critical appraisal of the methodological quality of selected guidelines (*e.g*., by using the AGREE instrument) [67].

As is common practice in other areas of research such as guideline development, the documentation of selection criteria for participating persons as well as the disclosure of their names and potential conflicts of interest could greatly add transparency to the whole development process and, as a result, increase the content validity of QIs.

### Extraction of recommendations

The main focus of this review is the extraction of guideline recommendations. This step is both crucial and unique to guideline-based QI development, whereas the other steps could also be applied to the development of QIs from other sources such as primary literature or existing QI sets. We only included studies that provided a detailed description of the recommendation-extraction process. As a result, we excluded a large number of otherwise eligible studies (see Additional file 4: Table 4 for a list of studies excluded for this reason).

The reviewed literature describes two different approaches to the extraction of guideline recommendations. The first approach is to initially extract all recommendations and to then select QIs using a systematic consensus process. The second approach is to select a limited number of recommendations during the extraction process. We believe the difference between both approaches is of crucial importance to the quality of ensuing QI sets. Predominantly, only a small number of persons conduct the extraction process. Often, those participants were not selected following transparent selection criteria. The extraction of potential QIs itself through this small group of participants usually does not follow any documented selection criteria, either. As a result, the final QI set may suffer from selection bias.

Subsequent systematic consensus processes to rate and select the extracted potential QIs are usually conducted by larger panels. In comparison to the small group of persons conducting the selection of potential QIs, panel participants are commonly selected to build a balanced panel of different professionals participating in the process of healthcare the QIs are developed for. In addition, the use of predesigned forms containing rating and selection criteria during these systematic consensus processes substantially reduces the risk of selection bias (see "QI selection").

Another important aspect of the extraction process is the translation of the guideline text into recommendations manageable as potential QIs. It can be difficult to derive appropriate numerators and denominators on the basis of the guideline recommendation wording, which may not be specific enough for this purpose. A whole paragraph of guideline text, for instance, cannot easily be translated into a potential QI without cutting out potentially relevant information. Thus, the translation process is a further potential source of bias.

Hence, both the selection of participants as such and the documentation of selection criteria for participants are of great importance. We identified a large deficit in the existing literature regarding this: Only five studies reported selection criteria for participants.

### QI selection

Panel methods are not specific to guideline-based QI development and are frequently used to systematically augment the evidence from guidelines with expert opinion (*e.g*., the widely used RAND/UCLA appropriateness method [68,69]). Performed carefully, this reduces the risk of unintentional influence of stakeholders on the results of the development process [70]. Panel methods are an established component of the development process of high-quality guidelines. As our results confirm, they are also widely used in the development of QIs [65]. Many of the reviewed studies showed a lack of transparency regarding the nomination process (*e.g*., in not providing explicit selection criteria for panel members).

Our results show that patient participation during QI development is extremely uncommon. In principle, the frequently used panel method offers room for the participation of patients or patient representatives. However, to date, no standardized approach to patient participation during QI development exists. To fill this gap, our working group is currently conducting a systematic review of approaches to patient participation during QI development.

### Practice test

Practice tests prior to publication and usage of QIs are an essential step in evaluating validity, reliability, feasibility, and other important attributes of QIs (see Background). They are an integral part of any implementation strategy and an essential component of the quality loop [7,26]. The practice test in a study by Wollersheim *et al. *[10] showed that between 10% and 20% of the developed QIs were not measurable.

It could be argued that regular evaluations of the usage of QIs suffice. However, given the impact QIs can have from day one of their application (*e.g*., if used in pay-for-performance models [see Background]) and the fact that QIs are more widely accepted after an advance test, it is desirable that practice tests under "laboratory conditions" become established components of the development process.

### Implementation

The importance of implementation strategies is often referred to in the course of critical appraisal of guidelines [42]. As for guideline development, implementation strategies are indispensable for the real-life application of QIs [58]. Our results show that even though a wide variety of implementation strategies are reported, they are not always part of the QI development process. Given the importance of implementation, a thorough discussion and application of implementation strategies should be an integral part of a gold-standard QI development method.

### Strengths and limitations

To our knowledge, this is the first systematic review of methodological approaches to guideline-based QI development. This systematic review has been conducted following a rigorous methodological approach [71]. The identification of methodological approaches to each step of guideline-based QI development allows a detailed description and comparison of the development methods published so far. We summarized the available evidence from systematically retrieved literature to provide a comprehensive overview of guideline-based QI development.

A major limitation of this study is that we were not able to provide answers to review questions 2 and 3. The selected studies were very heterogeneous in type, in terms of the quality of reporting and in the methodological approaches to guideline-based QI development presented. Because we could not identify any studies comparing different methodological approaches to guideline-based QI development and no gold standard exists to compare the published methodological approaches to, we were not able to provide an evidence-based judgment on the methodological approaches identified. Hence, we were not able to determine whether any of the methodological approaches (as a whole or as single development steps) is "superior" to the others in its ability to generate high-quality QIs.

However, in describing the methodological approaches used by the different working groups developing QIs, we provide a basis for further research. This research should seek to determine which of these methodological approaches applied to individual steps of the development process are best suited to constitute a development pathway that generates the "best" QIs. In order to achieve this aim in view of limited resources, existing guideline developers network infrastructure (*e.g*., the G-I-N) should be used to cooperate and formulate a gold standard, as proposed by Blozik *et al. *[20].

## Conclusions

A wide variety of methodological approaches are described in the literature for guideline-based QI development. It remains unclear which method leads to the best QIs, since no randomized controlled or other comparative studies investigating this issue exist.

In presenting a comprehensive methodological overview, we provide a groundwork for further research leading to an evidence-based gold standard for guideline-based QI development.

## Competing interests

The authors declare that they have no competing interests.

## Authors' contributions

TK designed the study; performed literature search and screening, literature retrieval, and data extraction and interpretation; and wrote and revised the paper. EB contributed to the initial study idea, study design, and data interpretation; critically revised the article for important intellectual content; and read and approved the final draft. MS contributed to initial study idea, study conception and design, and data interpretation; critically revised the article for important intellectual content; and read and approved the final draft.

## Supplementary Material

Additional file 1 **Table S1: Medline Search Algorithm**. Click here for file

Additional file 2 **Table S2: Screened Institutional Websites**. Click here for file

Additional file 3 **Table S3: Data extraction form**. Click here for file

Additional file 4 **Table S4: Table of excluded studies**. Click here for file
