# Supplementary material for: Methods for the guideline-based development of quality indicators--a systematic review
Source: Implement Sci. 2012 Mar 21;7:21. doi: 10.1186/1748-5908-7-21 (PMC3368783; doi:10.1186/1748-5908-7-21)
Supplement: Additional file 1 — Table S1: Medline Search Algorithm. [file 1748-5908-7-21-S1.DOC]

## Medline search algorithm

| ***Quality indicators*** | ***Clinical guidelines*** | ***Development*** |
| --- | --- | --- |
| 1. quality indicator$.tw | 12. guideline$.tw | 31. develop$.tw |
| 2. quality criterion$.tw | 13. practice guideline/ | 32. and\11,30,31 |
| 3. quality measure$.tw | 14. practice guideline$.tw |  |
| 4. performance indicator$.tw | 15. clinical practice guideline$.tw |  |
| 5. performance measure$.tw | 16. recommendation$.tw |  |
| 6. outcome measure$.tw | 17. guidance$.tw |  |
| 7. outcome indicator$.tw | 18. directive$.tw |  |
| 8. audit.tw | 19. health service$ research.tw |  |
| 9. outcome assessment.tw | 20. evidence based medicine.tw |  |
| 10. process assessment.tw | 21. quality assessment.tw |  |
| 11. or\1-10 | 22. quality assurance.tw |  |
|  | 23. consensus technique.tw |  |
|  | 24. delphi technique.tw |  |
|  | 25. RAND.tw |  |
|  | 26. UCLA.tw |  |
|  | 27. RAM.tw |  |
|  | 28. RAND appropriateness method.tw |  |
|  | 29. consensus development/ |  |
|  | 30. or\12-29 |  |
