# Supplementary material for: Methods for the guideline-based development of quality indicators--a systematic review
Source: Implement Sci. 2012 Mar 21;7:21. doi: 10.1186/1748-5908-7-21 (PMC3368783; doi:10.1186/1748-5908-7-21)
Supplement: Additional file 2 — Table S2: Screened Institutional Websites. [file 1748-5908-7-21-S2.DOC]

## Screened institutional websites

| ***Institution*** | ***Language*** | ***URL (last checked 02.23.2012)*** | ***Literature*** |
| --- | --- | --- | --- |
| American Academy of Otolaryngology – Head and Neck Surgery | English | http://www.entnet.org/ | - |
| Australian Council on Healthcare Standards | English | http://www.achs.org.au/ | - |
| AGREE  Collaboration | English | http://www.agreetrust.org/ | - |
| American Heart Association | English | http://www.heart.org/ | - |
| Agency for Healthcare Research and Quality | English | http://www.qualitymeasures.ahrq.gov/ | - |
| American Medical Association | English | http://www.ama-assn.org/ | - |
| Institute for Applied Quality Improvement and Research in Health Care (AQUA-Institute) | German, English | http://www.sqg.de/startseite/index-en.html |  |
| American Society of Clinical Oncology | English | http://www.asco.org/ | - |
| Arbeitsgemeinschaft der wissenschaftlichen medizinischen Fachgesellschaften (AWMF) | German | http://www.awmf.org/ | - |
| Agency for Quality in Medicine | German, English | http://www.aezq.de/ |  |
| BQS Institut für Qualität und Patientensicherheit | German | http://www.bqs-institut.de/ | - |
| Canadian Cardiovascular Outcomes Research Team | English | http://www.ccort.ca/ | - |
| German College of General Practitioners and Family Physicians | German | http://www.degam.de/ | - |
| EQUAM-Foundation | German | http://www.equam.org/ | - |
| Guidelines International Network | English | http://www.g-i-n.net/ | - |
| Initiative for Maternal Mortality Programme Assessment (Immpact) | English | http://www.immpact-international.org/ | - |
| IQ Healthcare | Dutch, English | http://www.iqhealthcare.nl/ |  |
| The Joint Commission | English | http://www.jointcommission.org/ | - |
| The National Association of Statutory Health Insurance Physicians | German, English | http://www.kbv.de/ | - |
| National Committee for Quality Assurance | English | http://www.ncqa.org/ | - |
| National Institute for Clinical Excellence | English | http://www.nice.org.uk/ | - |
| National Primary Care Research and Development Council | English | http://www.medicine.manchester.ac.uk/primarycare/npcrdc-archive/index.cfm.htm | - |
| New Zealand Guidelines Group | English | http://www.nzgg.org.nz/ | - |
| Organisation for Economic Cooperation and Development | English, French | http://www.oecd.org/ | - |
| RAND Corporation | English | http://www.rand.org/ | - |
| Royal College of General Practitioners | English | http://www.rcgp.org.uk/ | - |
| Royal College of Physicians | English | http://www.rcplondon.ac.uk/ | - |
| Scottish Intercollegiate Guidelines Network | English | http://www.sign.ac.uk/ | - |
