# Supplementary material for: Methods for the guideline-based development of quality indicators--a systematic review
Source: Implement Sci. 2012 Mar 21;7:21. doi: 10.1186/1748-5908-7-21 (PMC3368783; doi:10.1186/1748-5908-7-21)
Supplement: Additional file 3 — Table S3: Data extraction form. [file 1748-5908-7-21-S3.DOC]

## Data extraction form

| *Item* | *Extracted information* | | | | | | | | | | | | | |
| --- | --- | --- | --- | --- | --- | --- | --- | --- | --- | --- | --- | --- | --- | --- |
| Design | Method paper | | | Topic paper | | | | | | Method + topic paper | | | | |
| Publication type | Not mentioned | | | Mentioned in full text | | | | | | Mentioned on title page | | | | |
| Duration of the study | Not mentioned | | | | | | Mentioned | | | | | | | |
| Funding | Not transparent | Public funding | | | | | | Private funding | | | | Both public / private | | |
| Topic selection | No criteria reported | | | | | | | Criteria reported | | | | | | |
| Target population / setting | Not reported | | | Reported | | | | | | Method paper | | | | |
| Panel method | Modified RAND / UCLA | Other | | | | | | Unclear | | | | No panel method | | |
| Selection criteria for panel members | Not reported | Reported | | | | | | Unclear | | | | No panel method | | |
| Panel members | Not mentioned | | | Mentioned | | | | | | No panel method or method paper | | | | |
| Selection criteria for persons, who extracted recommendations | Not reported | | | | | | | Reported | | | | | | |
| Extracting persons | Not mentioned | Mentioned | | | | | | Unclear | | | | Method paper | | |
| Development of QI from... | One guideline | | | More than one guideline | | | | | | Guideline(s) and other sources | | | | |
| Method for guideline selection | Not mentioned | | | Mentioned | | | | | | Unclear | | | | |
| Critical appraisal of guidelines | Not conducted | | | Conducted | | | | | | Unclear | | | | |
| Selected guidelines | Not mentioned | | | Mentioned | | | | | | Method paper | | | | |
| Extracted recommendations | All | | | | Selection | | | | | | Unclear | | | |
| Criteria for selection of recommendations | Not mentioned | | Mentioned | | | | | | Unclear | | | | | No selection |
| Potential indicators | Not listed | | | | | Listed | | | | | | | Method paper | |
| Selected indicators | Not listed | | | | | Listed | | | | | | | Method paper | |
| Sources of the single indicators | Not mentioned | | Partially mentioned | | | | | | Always mentioned | | | | | Method paper |
| LoE of underlying recommendations | Not mentioned | | | | | Mentioned | | | | | | | Unclear | |
| Assessment of QI using explicit criteria | Not mentioned | | | | | Mentioned | | | | | | | Unclear | |
| Practice test | Not mentioned | | Included | | | | | | Planned | | | | | Proposed |
| Implementation strategy | Not mentioned | | | | | | | | Mentioned | | | | | |
| Patient participation | No participation | | | | | Guideline selection / extraction | | | | | | | QI selection | |

QI = quality indicator; LoE = level of evidence.
