# Supplementary material for: Methods for the guideline-based development of quality indicators--a systematic review
Source: Implement Sci. 2012 Mar 21;7:21. doi: 10.1186/1748-5908-7-21 (PMC3368783; doi:10.1186/1748-5908-7-21)
Supplement: Additional file 4 — Table S4: Table of excluded studies. [file 1748-5908-7-21-S4.DOC]

## Table of excluded studies: Exclusion criterion: Recommendation-extraction process not detailed

| **Author** | **Publication** | **Topic** |
| --- | --- | --- |
| Bilimoria *et al.* 2009 | *Journal of the National Cancer Institute* | Pancreatic cancer care |
| Charbonneau *et al.* 2004 | *Medical Care* | Depression care |
| Craft *et al.* 2000 | *The Medical Journal of Australia* | Breast cancer care |
| DiSalvo *et al.* 2001 | *American Journal of Medicine* | Cardiovascular care |
| Flanagan *et al.* 2007 | *Medical Care* | Prevention and control of AMR |
| Guru *et al.* 2005 | *Journal of Thoracic and Cardiovascular Surgery* | CABG surgery |
| Hakonsen *et al.* 2006 | *Pharmacy World & Science* | Cancer pain management |
| Harr *et al.* 1996 | *American Journal of Medical Quality* | Depression in primary care |
| Hermann *et al.* 2006 | *International Journal for Quality in Health Care* | Mental healthcare |
| Horongo *et al.* 2005 | *International Journal for Quality in Health Care* | Tuberculosis services |
| Huang *et al.* 2010 | *Quality and Safety in Health Care* | Drug therapy |
| Jacobsen *et al.* 2007 | *Journal of Oncology Practice* | Treatment of colorectal cancer |
| Ko *et al.* 2008 | *Canadian Journal of Cardiolology* | Percutaneous coronary interventions |
| Malafa *et al.* 2009 | *Cancer Control* | Cancer care |
| Mangione-Smith *et al.* 2007 | *New England Journal of Medicine* | Ambulatory care delivered to children |
| Mourad *et al.* 2008 | *Human Reproduction* | Subfertility care |
| Radley *et al.* 2001 | *Pharmacy World & Science* | Delivery of pharmaceutical care |
| Saag *et al.* 2004 | *Arthritis & Rheumatism* | Arthritis care |
| Sampsel *et al.* 2007 | *Clinical and Experimental Rheumatology* | Arthritis and osteoporosis care |

AMR = antimicrobial resistance; CABG = coronary artery bypass graft.
